# A Volume-Tuning Capillary Gripper That Enhances Handling Capabilities and Enables Testing of Micro-Components

**DOI:** 10.3390/mi13081323

**Published:** 2022-08-16

**Authors:** Adam Chafaï, Amin Ibrahimi, Pierre Lambert

**Affiliations:** 1 Transfers, Interfaces and Processes (TIPs) Department, Université Libre de Bruxelles (ULB), CP 165/67, 50 Av. F.D. Roosevelt, 1050 Brussels, Belgium; 2 Department of Mechical Engineering, Vrije Universiteit Brussel (VUB), Bd. de la Paine 2, 1050 Brussels, Belgium

**Keywords:** micro-assembly, micro-manipulation, gripper, capillary force, capillary transportation, pick-and-place, semi-conductors

## Abstract

Capillary forces are shown to be extremely effective for micro-assembly and pick-and-place processes, especially for their ability to self-align the handled objects. However, in today’s machines, micro-objects are submitted to high loads, such as compressions for the electrical testing of the micro-components, or inertial forces coming from the high accelerations of the machines. There, capillary grippers may show some limits. These issues, as well as the difficulty to perform precise visual inspections (due to the tilt of the handled micro-object that can occur after a perturbation, such as the displacement of the gripper), can all be solved by temporarily removing the liquid meniscus. Therefore, we present a novel volume-tuning capillary gripper that provides a solution to these limitations without adding additional significant complexities or changes to the existing pick-and-place machines. A multi-scale prototype was dimensioned and produced by using fast prototyping methods, such as a femtosecond laser-assisted chemical etching process for fused silica. Models bringing a deeper understanding of the subsystems are presented. The proof of concept was extensively tested. Its picking capabilities and enhancements of the handling capabilities during horizontal motions, as well as the repeatability of the tuning of the volume of liquid, are presented.

## 1. Introduction

Handling objects at and below the millimeter scale is a challenging process. Pick-and-place machines (currently mainly using vacuums as gripping principles to handle and test the micro-components at the end of their manufacturing processes) start showing some limits when the micro-components reach a few hundreds of micrometers. These limits are due to the constraints of the number: in some cases, one high-speed pick-and-place machine can have to process 1 million components a day; 12 parts a second, per machine. The machine must perform visual and electrical inspections, looking for defects. Pick-and-place machines, which put together the micro-components in the final product, are also concerned by this constraint of operating at high rates. Reaching such rates is only possible by means of high accelerations, up to dozens of times the gravitational acceleration. Vacuum grippers operate well at these accelerations.

Among all the other handling principles (acoustic levitation, friction gripping, etc.) [1], capillary grippers (exploiting the surface tension forces of the meniscus of a liquid to handle the micro-objects) are shown to be promising candidates to replace the market-leading vacuum technology. However, they are not yet mature enough in their current forms when considering high dynamics pick-and-place processes. Capillary forces rely on the effect of surface tension to create an elastic liquid bond between two objects (here, the gripper and the component to handle). The capillary force can be described as the sum of two terms: the Laplace force, which depends on the curvature of the liquid joint, and can either be attractive or repulsive; and the tension force, which pulls on the triple line interface, tangentially to the latter. The tension force is always attractive.

The primary idea of exploiting capillary forces to handle micro-objects dates back to the works by Bark et al. [2] and Grutzeck et al. [3]. One objective of the capillary gripper is to take advantage of the self-alignment effect, to obtain a micrometric precision on the placement of the components. The self-alignment effect relies on the capillary forces that are opposed to the deformations of the meniscus. The result is that, in the right wetting conditions, the equilibrium position (aligned and centered with the gripper) will be retrieved after a displacement of the component [4]. No need to use precise positioning actuators anymore. To achieve this, lubrication by the liquid meniscus is necessary. However, this lubrication layer is counterproductive when it comes to fighting high inertial forces: dry friction would help to avoid the loss of the micro-object. Cherville et al. [5] proposed. a solution to enhance the handling capabilities by exploiting a vertical wall to generate a reaction force opposing the inertial force.

The work by Zhang et al. [6] let us imagine that the permanent friction and the reaction forces that their capillary gripper can deploy could be an asset concerning the handling capabilities.

Even though these grippers have not been designed with this transport issue in mind (most of the time, they are aimed at changing the intensities of the capillary forces for the release of the components), the literature provides us with leads and objects of reflection to create a gripper optimized for the lateral transport phase. The tuning of the capillary forces can be obtained by monitoring the wettability. For instance, capillary forces can be tuned thanks to the electro-wetting effect [7,8,9]. A gripper made of a rough elastic membrane able to modify its wettability by stretching was proposed by Fantoni et al. [10]. Based on Israelachvili’s model [11], Biganzoli et al. [12] and Pagano et al. [13] investigated the changes in conformity of their grippers thanks to inflatable membranes, distorting their grippers from flat to hemispherical. Such mechanisms allow decreasing the gripping forces and, therefore, releasing the picked objects. Iazzolino et al. [14] have shown that the tuning of the capillary forces can be obtained by tuning the volume of the liquid meniscus. In this case, the objective is the release by evaporation, based on a change in the contact conformity that decreases the capillary forces. Lambert et al. [15], in whose work the conical cavity of the gripper was used to generate larger capillary forces, also exploited the conformity concept and enhanced the gripping capabilities. In addition to being a limitation during transportation, the presence of a liquid layer is also an issue in two other situations:

1. In pick-and-place processes, components must be visually inspected for any structural defects. For this operation, the horizontality default of the component under the gripper must typically be below $5^{\circ }$, or the component’s shape will be too distorted (due to the perspective) and discarded by the machine. This is of course no problem with the vacuum grippers, where the dry and flat contact ensures the horizontal positioning. However, for liquid menisci, as the wedge configuration is found to be stable [16], the horizontal configuration may not be obtained after a perturbation such as a high acceleration. Depending on the volume of the liquid meniscus, it may yield wedge angles above $5^{\circ }$.
2. Some components must also be electrically tested by compressing on micro-connecting terminals, after having been aligned correctly. The typical pressure to be applied by the gripper on the component is 2 N·mm${}^{-2}$. For a regular passive capillary gripper, the lubrication layer would be squished, and the liquid would overflow, disrupting the rest of the process.

A solution is to design a system that can produce the liquid meniscus (to produce the self-alignment effect) and remove it when required (for transportation, tests, inspections, etc.). Many authors have explored volume-tuning solutions for the generation of droplets on their grippers. However, they have not considered the possibilities it offers in terms of the handling capabilities, or testing of the micro-components.

A widely used solution involves relying on pressure or volume controllers [2,3,15,17], or on a Peltier element that can be used to control the volume of liquid by condensation and evaporation [18].

However, these solutions present a drawback: they depend on the responsiveness of their actuators to create and remove the liquid menisci, which can limit the production rate of the machines. Moreover, removing vacuum gripper heads from the system to add new liquid volume controllers may sound counterproductive in terms of cost and complexity.

This work was driven by the need to solve the previously mentioned issues, (handling capabilities, visual inspection, and electrical testing) by proposing a novel passive handling solution. By this, we mean a system where no additional actuator would be required besides existing positioning ones. This capillary gripper was designed to exploit the structural elasticity of flexible sub-systems to control the available amount of liquid. One advantage of using such flexible sub-systems, which will be presented later, is that all hysteresis sources are removed from the design, allowing its open-loop actuation, and increasing its precision.

The passive capillary gripper that will be presented here can be filled with liquid in one simple operation and can pick components by dispensing a controlled amount of this liquid.

With this design, the amount of liquid remains zero while the gripper moves to enhance the handling during the transport phase. Once in the lower position, the volume of the liquid can be tuned by using the vertical actuator. As will be shown later, this alone allows the capillary gripper to correctly perform visual and electrical tests.

## 2. Sequential Actuation

**Figure 1:**
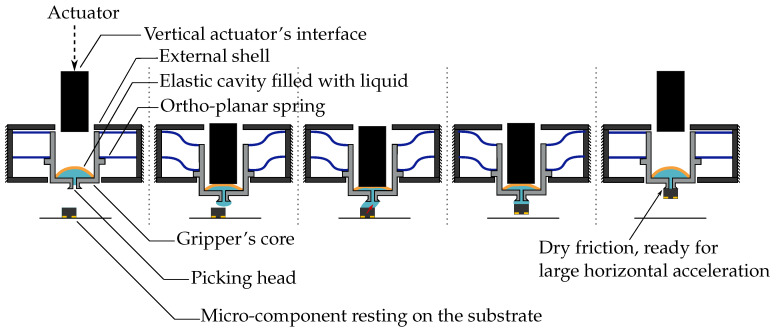
Schematic overview of the passive capillary gripper. The gripper and its guided interface can be mounted in a pick-and-place machine. By applying pressure with the vertical actuator on the interface, the gripper is moved downwards. The latter is guided by two ortho-planar springs in parallel. The stiff cavity holds the load of the interface and transmits it to the two springs, which deflect. Once the stops are reached, the cavity deflects, which generates a droplet used to pick the component. The volume of the droplet can be controlled by changing the pressure applied by the pick-and-place machine’s vertical actuator.

As presented in Figure 1, the capillary gripper consists of one external shell relative to which a vertical actuator’s interface can move. The height of the shell represents the working height of the capillary gripper. The actuator’s interface is a secondary guiding system that allows making this passive capillary gripper compatible with the existing positioning systems. It is made based on the concept of double parallelogram beams as studied by Patil et al. [19]. A view of the vertical actuator will be shown in Section 4.

The machine’s vertical actuator meets this interface to move the gripper’s core. There, the actuator’s interface meets a stiff cavity and exerts pressure on it. In a competition of stiffnesses $k_{c}$ and $k_{\mathrm{OPS}}$, respectively, the cavity and ortho-planar spring stiffnesses, we aimed to have minimal compression of the cavity during the vertical motion, i.e., most of the stress must be transmitted to the two parallel ortho-planar springs.

**Figure 2:**
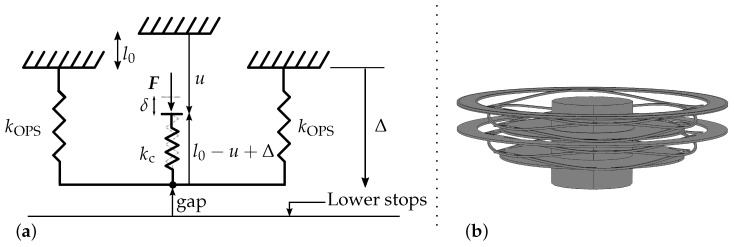
(**a**) Mechanical representation of the stiffnesses competition between the ortho-planar springs in parallel and the cavity. (**b**) Two elongated ortho-planar springs in parallel forming a linear guiding system. The outer boundary of the springs is clamped.

To ensure a total stroke Δ, a force *F* must be applied to the vertical actuator’s interface, so that *F* yields a compression of the cavity $\delta =\left|\right|\left(l_{0}-u+\Delta \right)-l_{0}\left|\right|=u-\Delta$, where *u* is the general compression height of the system and Δ is the elongation of the ortho-planar springs (see Figure 2a). The force *F* to be applied to the system writes:

(1) $$F=k_{c}\left(u-\Delta \right)=2k_{\mathrm{OPS}}\Delta ,$$

and the resulting compression height *u* writes:

(2) $$u=\Delta \frac{2k_{\mathrm{OPS}}+k_{c}}{k_{c}},\;\mathrm{and}\;u^{*}=\Delta_{\max}\frac{2k_{\mathrm{OPS}}+k_{c}}{k_{c}},$$

when the lower stops are reached, i.e., the gap is zero (typically, $\Delta_{\max}=0.85$ mm, it is enough to work at a reasonable distance from the potential obstacles).

As we want Δ≫δ, as long as the lower stops are not met, the vertical stiffness of the cavity $k_{c}$ must be large, relative to the stiffness of ortho-planar springs $k_{\mathrm{OPS}}$.

This internal degree of freedom, represented in Figure 2b, will guide the gripper’s core to its low position, where it will meet the lower part of the external shell that will block the motion. As the shell and the stops are considered rigid in comparison with the cavity, the pressure applied by the actuator will deform the membrane and dispense the liquid.

Similarly, the upper part of the shell is used to stop the ascending motion of the ortho-planar springs, in order to perform the electrical compressive tests. Therefore, the shell is designed to hold the loads of the vertical actuator and the micro-connection terminals.

## 3. Design Overview and Assembly Process

**Figure 3:**
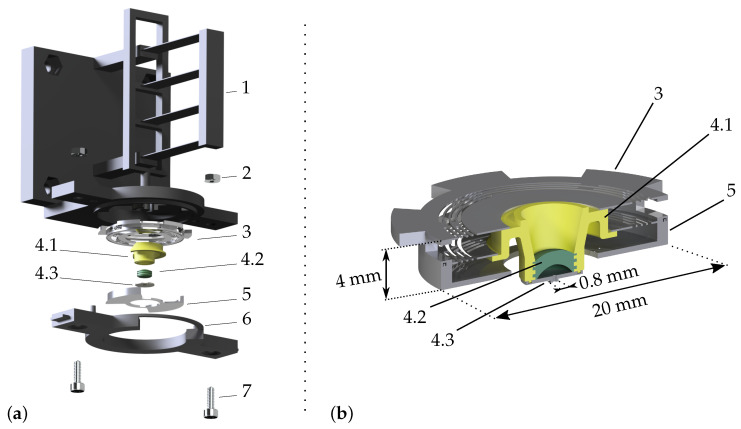
CAD representation of the manufactured parts. (**a**) Exploded view of the system with the order of assembly. The gripper’s core (the body 4.1 in which the cavity 4.2 was molded and closed by the micro-metric picking head 4.3) was assembled with the monolithic parallel ortho-planar springs 3 and enclosed by the lower stops 5. The system was sealed by screwing the lower casing 7 on the interface 1. (**b**) Cut view of the gripper’s core (4.1, 4.2, 4.3) attached to the ortho-planar springs 3 and the lower stops 5.

As this design is a multi-scale design, the manufacturing of the prototype was performed in several steps (with different techniques) and assembled. Figure 3a shows the manufactured parts with the order of assembly.

A closer view of part numbers 3, 4.1, 4.2, 4.3, and 5 is shown in Figure 3b.

The parts were assembled with the gripper upside down so that the parts could lay on each other during the assembly. Interface 1 is composed of compensated parallel beams as discussed in Section 2. The upper stops used to block the ascending motion of the gripper during the compressive electrical test (see Section 6.4) are part of the interface. Note that the choice of design made for the vertical actuator’s interface ensures a quasi-purely vertical application of the load on the membrane. This guiding system indeed presents a minimal parasitic deflection in the orthogonal directions, which therefore minimizes the radial stress transmitted to the ortho-planar springs. The parallel ortho-planar springs 3 were placed on 1. The gripper’s core 4 was mounted in the center of 3. These parts were assembled using UV glue.

The gripper’s core is made of a body 4.1, in which membrane 4.2 was molded thanks to its two negative molds (not represented). Grooves in the body keep the membrane in place and ensure the water tightness of the cavity.

The cavity was then closed by the micro-metric picking head 4.3 using UV glue.

The lower stops 5, on which the gripper’s core is compressed to generate the droplet of liquid, was assembled with 4 and glued. The system was sealed by screwing the lower casing 6 on the interface with the screws 7 and bolts 2.

## 4. Manufacturing Techniques

Considering the different requirements for each part, different manufacturing choices were made. They are presented in Table 1 along with the requirements that led to these choices.

### 4.1. Manufacturing of the Fused Silica Parts

**Figure 4:**
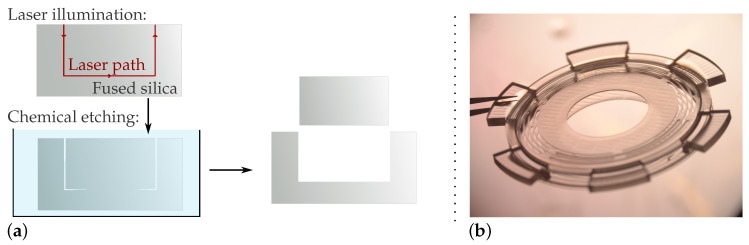
(**a**) Illustration of the subtractive printing process for fused silica. The fused silica was locally illuminated by a femtosecond laser, which increased its etching rate. By illuminating complete areas, blocks of material can be detached and the desired structure can be obtained. (**b**) Final prototype of the monolithic parallel ortho-planar springs after etching (outer radius: 10 mm, thickness of the springs: 0.144 mm).

Manufacturing of the fused silica parts 3, 4.3, and 5 was performed by means of a femtosecond laser-assisted chemical etching process. The laser was used to locally illuminate the glass. This operation alters the material, which becomes more easily etchable in acids or bases (typically highly concentrated hydrofluoric acid or potassium hydroxide solutions). By illuminating complete areas, blocks of fused silica can be detached in order to produce the desired part. Figure 4a illustrates the laser illumination and etching processes.

This process allows precision at the micrometer scale and the rapid manufacturing of complex monolithic parts. Fused silica is an excellent material for thin flexible structures. It is a perfectly elastic material that does not yield. With its Young’s modulus of 72 GPa, the material is as rigid as aluminum. Its fragile behavior involves that the characterization of the structure must be rigorously performed and that strong safety coefficients must be taken. Figure 4b shows the final prototype of the monolithic parallel ortho-planar springs after etching (outer radius: 10 mm, thickness of the two springs: 0.144 mm).

The shape of the final prototype, presented in Figure 4b, was optimized to ensure a uniform chemical etching and the correct clamping of the springs’ outer radii. A cut view is shown in Figure 3b. After manufacturing, careful observations with a confocal microscope were performed in order to measure the thickness of the springs and the size of their features. These measurements were input in the numerical and analytical models presented in Appendix A.1, and used for the characterization of the system presented in Section 6.

### 4.2. Micro-Molding of Silicone Rubber in SLA Molds

The body 4.1 along with the two molds required to create the membrane were printed with a stereolithography machine. However, most UV-sensitive liquid resins contain compounds that will inhibit the curing of silicone rubbers. As the curing inhibition effect is limited when the resin is fully polymerized, the walls were made thin enough to minimize the volume of resin to post-cure. A close view of the part is available in Figure 3b. Furthermore, a transparent resin was used, and the parts were designed to allow the UV light to reach most of the surfaces by considering conical shapes instead of cylindrical ones. The printed parts were properly rinsed out with isopropyl alcohol and water before being dried and overexposed to UV light (exposure time: 30 min). The parts were then coated by misting several isolating layers of transparent mat finish acrylic varnish on all the surfaces. The two molds only were sprayed with a release agent.

A tin cure silicone rubber with a Shore-A 40 hardness was used to create the deformable cavity, as tin cure rubbers are less sensitive to curing inhibition agents than the platinum cure ones. The two compounds (the rubber base and curing agent) were mixed by weight (1:10 ratio). After a droplet of silicone was pipetted in the mold, the latter was left open and degassed. The mold was then closed and left to cure for a minimum of 16 h.

To avoid air trapping while closing the mold, the droplet of resin was deposited in the body, on the concave part of the mold before degassing, and the mold was then closed with the convex part. Cavities (with thicknesses of 0.5, 1, and 2 mm) were manufactured this way, with no curing issues.

## 5. Materials and Methods for the Characterization of the Capillary Gripper

The gripper’s characterization and validation of the proof of concept were performed on a test bench that includes a module for the filling of the cavity, in which a droplet of pure water was deposited with a micro-pipette to control the volume. Two cavity-filling procedures were considered (the module could be tuned in height for one of them). The procedures are explained in detail in Section 6.1.

At each cycle, a component resting on a transparent substrate was picked by the gripper. The latter was guided over the test bench by a linear stage. It was actuated vertically by a second linear stage, applying pressure on the actuator’s interface. Below the transparent substrate, a 45${}^{\circ }$ mirror, in conjunction with a side view camera, allowed complete observation of the picking and self-alignment motions.

A compression module (presented in Appendix B) allowed the application of a known load on the component handled by the gripper. In this way, a pressure of 2 N·m${}^{-2}$ could be applied to the component.

As it is reasonable to consider that the release will be operated by a feature of the pick-and-place machine (shearing in a carrier tape, soldering on an electronic card, release on a vacuum nest, or an adhesive wafer), this point was not studied further: the release was performed here on an adhesive substrate for illustrative purposes. Except for the shearing in the carrier tape, one should note that these release strategies preserve the alignment provided by the surface energy minimization of the liquid meniscus.

Note that a residual amount of liquid is transferred to the component when the liquid bridge breaks.

## 6. Results and Discussion

### 6.1. Characterization of the Silicone Cavity

#### 6.1.1. Stiffness of the Cavity

**Figure 5:**
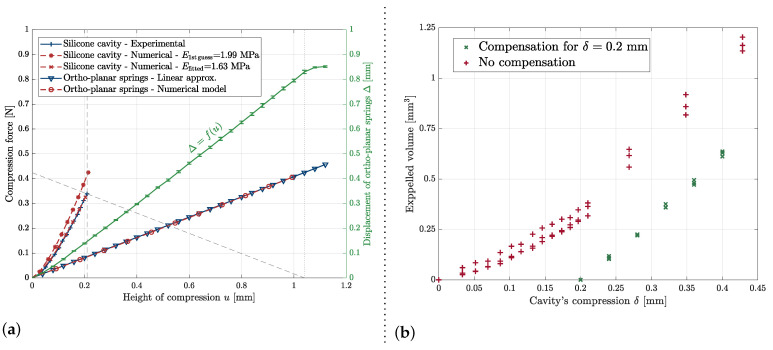
(**a**) Results of the experimental characterization of the cavity. The known stiffnesses of the ortho-planar springs were used to determine the force characteristic of the elastic cavity. This force characteristic was compared with the solution of a finite element model (presented in Appendix A.2). (**b**) Expelled volume as a function of the cavity’s compression δ. The figure shows the expelled volume for no compensation of the parasitic compression before the stops were reached and with compensation.

The ortho-planar springs, which are mounted in series with the elastic cavity according to Figure 2, have known stiffnesses. The latter is obtained by modeling this subsystem. Two models are presented in Appendix A.1. One uses a finite element method and includes 3D geometrical non-linearities, while the second is a 2D simplification for which an analytical expression is proposed for small elongations. The stiffness of the cavity can therefore be experimentally determined by varying the compression height *u* and measuring the axial elongation of the ortho-planar springs Δ. For a given compression height $u_{i}$, the duty point of the system is given by the intersection of $u=u_{i}-\Delta$ and $F=-2k_{\mathrm{OPS}}u+2k_{\mathrm{OPS}}u_{i}$. The intersection of the dashed lines in Figure 5a shows the duty point for $u_{i}=1.04$ mm, yielding an elongation of the ortho-planar springs Δ=0.83 mm. *u* is incrementally changed, 40 μm at a time, and Δ is tracked thanks to an algorithm based on Shi et al.’s tracking algorithm [20]. The experiment was reproduced three times; the averages and the standard deviations for each value of *u* are displayed in Figure 5a.

The experimentally-obtained force–compression characterization for the cavity is compared with the solution of a 2D axisymmetric finite element model presented in Appendix A.2. Our first guess for the Young’s modulus of the silicone (1.99 MPa) is thus assessed. A more satisfying fit was obtained for $E_{\mathrm{fitted}}=1.63$ MPa, which gives a relative error of 18% relative to our first estimation from an empirical model.

#### 6.1.2. Tuning of the Liquid Volume by Controlling the Vertical Positioning Actuator

The silicone cavity is filled by compressing it between the actuator’s interface and the lower stops. However, as developed in Section 2, the silicone cavity undergoes a parasitic compression as the gripper’s core is moved towards the component. As this effect might require avoidance, in this section, we propose a strategy to avoid the generation of a parasitic droplet during the vertical motion of the capillary gripper.

With a cavity full of liquid, a compression would result in the generation of a droplet during the motion of the gripper’s core towards the component. Although this parasitic dispensed volume does not prevent the proper use of the gripper, it might be unintended in some cases.

To compensate for the parasitic compression, the possibility of allowing a certain volume free of liquid was studied. In this case, the cavity was compressed, touched the mother droplet on the substrate, and was then decompressed until a certain point to suck in the liquid. Then, the liquid bridge between the mother droplet and the gripper was broken by moving the mother droplet down, and the cavity was fully decompressed. By doing so, the gripper sucked in a volume of air. The latter was released first at the next compression, and no parasitic droplet was observed. In practice, Figure 5a shows that compensation for a parasitic compression of δ = 0.2 mm should prevent parasitic droplets.

The liquid generation was experimentally studied by following this sequence. The experimental generation of droplets is shown in Figure 5b. The experiment was repeated three times by increasing the height of compression *u* with a pitch of 40 μm, and the repeatability was satisfactory. No parasitic volume was observed before the lower stops were reached.

To characterize the volumes accessible over the full range of compressions δ, the experiment was also conducted without compensating for the parasitic volume (see Figure 5b—“No compensation”). The experiment was repeated three times.

In conclusion, the gripper can be filled, and a controlled and precise volume of liquid can be generated by applying the right over-pressure with the actuator once the lower position of the gripper is reached. When the gripped is refilled during the process, the same volume can be obtained by applying the same pressures, which makes the liquid generation predictable and repeatable. Although we believe the parasitic liquid generation described earlier is not an issue for the process, it can be compensated by only partially filling the cavity.

### 6.2. Picking and Self-Alignment of the Components

To show the gripping capabilities of the gripper, square components of varying sizes and masses were considered. The meshing of the experimental space was obtained by varying the thickness of the components for a given surface. These components were made by laser assisted chemical etching of fused silica, and 24 different components were manufactured.

Before each trial, the gripper was positioned above the component with a miss-alignment representing 30% of the component’s size. The working distance was 2 mm. Note that the latter is defined as the distance between the top of the component and the gripper head at rest, i.e., when no pressure is applied by the vertical actuator.

The test is composed of assessing whether the component can be picked with the considered working distance and whether it is self-aligned.

Note that the working distance strongly influences the gripper’s capabilities to pick components: the smaller the working distance, the higher the picking forces. However, the choice of the 2 mm gap was based on the fact that the gripper should keep a safe distance from the ground and the potential obstacles when it is horizontally moved.

One should also note that the picking head is square. This allows controlling the alignment of the rectangular component modulo $90^{\circ }$ through the surface energy minimization of the liquid meniscus, allowing two equilibrium configurations.

**Figure 6:**
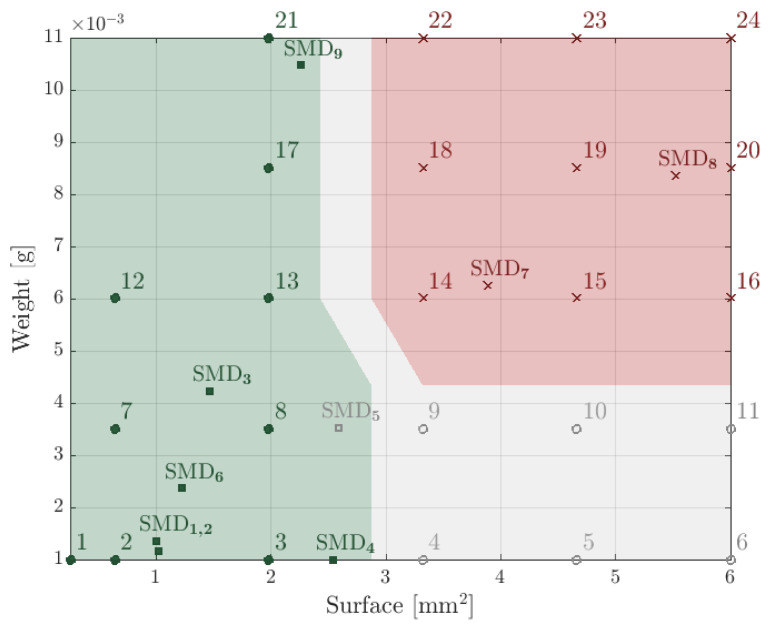
Results of the picking tests. Fused silica components (numbered from 1 to 24) and surface-mounted devices (numbered from SMD${}_{1}$ to SMD${}_{9}$) of varying surfaces and weights were tested. The green area represents a successful picking and self-alignment. In the gray area, the picking occurred but the self-alignment did not. The red area codes for the failure of both the self-alignment and the picking.

Figure 6 shows the results of the picking tests. The meshing of the experimental space is materialized by the glass components ranging from 1 to 24, the surfaces and masses of which can be read on the axes. The green area represents the dimensions for which the picking and self-alignment were successful for the glass components; the gray area codes for the dimensions for which only the picking occurred, and finally, the components in the red area were not self-aligned nor picked. The components at the border between the areas were tested three times each to assess the repeatability. For each one of the tested components, the conclusions were the same for each trial.

A clear boundary is visible, and the results are consistent with the physics they arise from. The smallest and lightest components were picked. Increasing the surface at a constant weight will result in partial wetting on the surface and, thus, in no self-alignment. Larger weights will overcome the capillary forces, resulting in no picking, although this was not observed for the smallest areas, where increasing the weight did not result in a picking failure. However, further tests in an expanded experimental space (with heavier components) might yield these expected results for the smallest components.

In addition, surface-mounted devices were tested and superimposed to the results of the fused silica components. In the case of SMD${}_{5}$, on the boundary between the green and gray areas, wetting defects yielded self-alignment failure. SMD${}_{5}$ is indeed an overmolded component with a surface made of epoxy. While the edges of SMD${}_{4}$ (made of external metallic electrodes) were sufficiently wetted to cause its alignment, the edges of SMD${}_{5}$ were not reached by the liquid during the picking, causing an alignment failure (a criterion for the success of the self-alignment is presented in the work of Mastrangeli et al. (2017) [4]).

To better evaluate these results, one should finally note that we present the picking and self-alignment capabilities for one size of the gripper head. Given its area (0.8×0.8 mm${}^{2}$), the gripper is better suited for components of approximately the same size. Therefore, it shows limits for much larger and much smaller components. However, the design of the gripper head can easily be adapted to operate with components of other sizes.

### 6.3. Components Transportation

This section presents qualitative results highlighting the enhanced handling capabilities of the passive capillary gripper. There, the gripper was used in two configurations. In the first (and intended) configuration, the component is picked, and the actuator relieves the pressure it applies to the system. The liquid is therefore sucked into the cavity, and the capillary forces at contact (in addition to the suction exerted by the silicone cavity in the case of a potential lack of liquid) create a friction force that acts against the inertial force induced by the horizontal motion of the gripper.

In the second configuration, constant pressure is kept on the membrane during the horizontal motion, resulting in a thin layer of liquid between the gripper and the component. This layer was made as thin as possible to have the highest lateral restoring forces, without friction between the component and the gripper head. This configuration aimed at mimicking a simple passive capillary gripper with no cavity, where the liquid meniscus would therefore need to be present during the whole process to ensure the self-alignment effect. Note that, in this case, no friction could occur and only the capillary forces acted against the inertial force.

**Figure 7:**
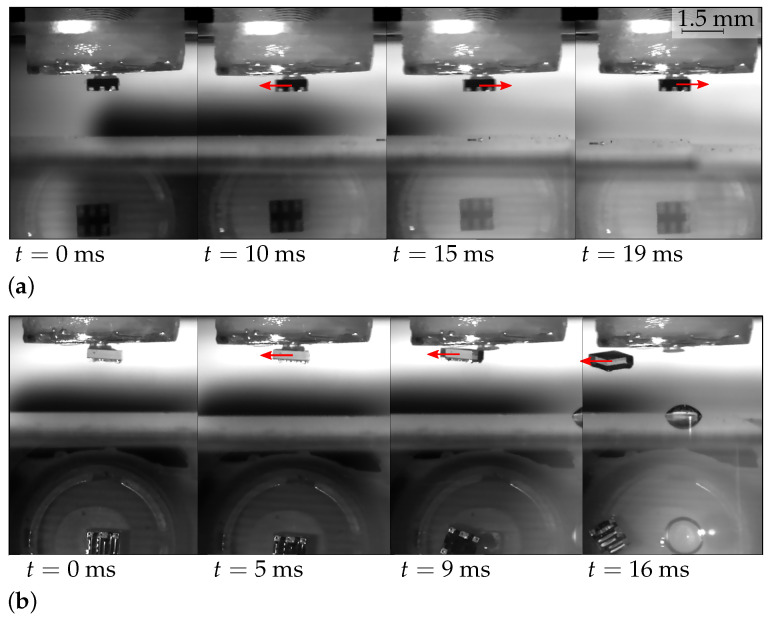
Successive frames of the transportation tests (acceleration and deceleration peaks: 44 m·s${}^{-2}$). The arrows depict inertia. (**a**) The passive gripper is used as intended, so that friction occurs between the component and the picking head when the pressure of the vertical positioning actuator is relieved. In this case, the component remains attached to the gripper and shows no motion relatively to the latter; (**b**) In order to mimic a standard passive capillary gripper where a thin layer of liquid is present at all times to self-align the component, a constant pressure is kept on the cavity. The presence of the liquid layer limits the handling capabilities.

Figure 7a,b show the comparisons between the results of these two configurations. The red arrows in each frame depict the inertial force directions. In the lower half of the pictures is shown the gripper seen from below, by reflection on the $45^{\circ }$ mirror, through the transparent substrate.

For the same acceleration profile (with acceleration and deceleration peaks of 44 m·s${}^{-2}$), the results strongly differ. In Figure 7b, the component was lost backward (during the acceleration), due to too low lateral restoring forces and the absence of friction. In Figure 7a (i.e., where the handling capabilities were enhanced by taking advantage of the gripper’s design) the component did not move relative to the gripper during the acceleration, nor during the deceleration.

Although this test highlights the handling enhancement induced by our design, compared to a standard capillary gripper without control of the liquid meniscus volume, it does not quantitatively characterize the maximal handling capability of the gripper, and further developments should focus on these quantitative tests.

### 6.4. Compression of Components

The component’s compression against the gripper was tested thanks to a compression module made of a table guided by two parallel deformable beams. The test was composed of picking the component, self-aligning it, and compressing it with a pressure of 2 N·mm${}^{-2}$. Eventually, the meniscus was regenerated by compressing the cavity, to proceed to further alignment operations.

Details on the compression module are available in Appendix B.

### 6.5. Proof of Concept Videos

A video showing the elongation of one ortho-planar spring made of fused silica is available in the supplementary material. This differently-scaled version (smaller in diameter and thinner) is representative of the kinematics of the design discussed in the article.

The compression test, together with the other steps of the process (droplet generation, picking, self-alignment, transport, and an example of release), are presented in the proof of concept video, available in the supplementary material.

## 7. Conclusions

A passive capillary gripper designed to address unanswered concerns about capillary grippers by providing a novel technical solution was presented.

This gripper was designed to create a passive system (no additional actuator, no additional controller on the pick-and-place arm) able to enhance the handling capabilities of capillary grippers. Although the tests performed show the difference between standard passive capillary grippers and this novel design in terms of handling capabilities, further work could focus on providing a finer characterization by varying the inertial force and assessing the limitations of the gripper in terms of handling capabilities.

Common passive capillary grippers would require presenting a liquid meniscus at all times in order to self-align a component in one particular step of the process. The design presented here allows the withdrawal of the liquid once the self-alignment step is performed, thanks to a compressible cavity. This passive mechanism allows operations which had not been demonstrated to be possible so far for capillary grippers (e.g., electrical testing of components by compression, visual inspection with a systematic horizontal positioning of the micro-object).

The passive aspect of the design is essential, as it implies that the already existing horizontal and vertical positioning actuators of the machine control:

- The horizontal positioning;
- The vertical displacement of the gripper;
- The generation of the volume of liquid, making possible the visual inspections and compressive tests, and enhancing the handling capability by creating friction;
- The alignment of the component under the gripper.

The design was manufactured with different techniques, depending on the requirements (FDM, SLA, micro-molding, subtractive manufacturing of fused silica parts). These multi-scale parts were assembled to create a working proof of concept.

The functions of the gripper were tested, including the compression. The enhancements of the handling capabilities were highlighted by a comparative test. Quantitative experimental results were delivered for the picking and the self-alignment of components, and the generation of liquid from the compression of the cavity. The ortho-planar springs and the cavity were modeled numerically in order to dimension the prototype. A 2D analytical simplification of the ortho-planar springs was proposed for the axial actuation and the range of validity was discussed.

## Figures and Tables

**Table 1:** Overview of the manufactured parts and their respective manufacturing techniques. The choice was based on the footprint of the parts (i.e., their overall size), the accuracy needed for certain features, and certain mechanical specificities, such as superior elastic properties.

Part Number	Footprint	Required Accuracy	Required Specificities	Manufacturing Technique
1	$10^{-2}$ m	$10^{-4}$ m	Printing of long and thin layers in suspension	Multi-material fused deposition modeling (FDM, tough PLA & PVA)
2	–	–	–	–
3	$10^{-2}$ m	$10^{-6}$ m	Excellent elastic properties, complex structure	Femtosecond laser-assisted chemical etching of fused silica
4.1	$10^{-2}$ m	$10^{-5}$ m	Compatibility with the molding process	Stereolithography (SLA) and coating protecting against curing-inhibition of silicone
4.2	$10^{-2}$ m	$10^{-5}$ m	Low Young’s modulus	Micro-molding of silicone from SLA molds
4.3	$10^{-3}$ m	$10^{-6}$ m	High Young’s modulus	Femtosecond laser-assisted chemical etching process of fused silica
5	$10^{-2}$ m	$10^{-5}$ m	High Young’s modulus	Femtosecond laser-assisted chemical etching process of fused silica
6	$10^{-2}$ m	$10^{-4}$ m	–	FDM (Tough PLA)
7	–	–	–	–

**Figure A1:**
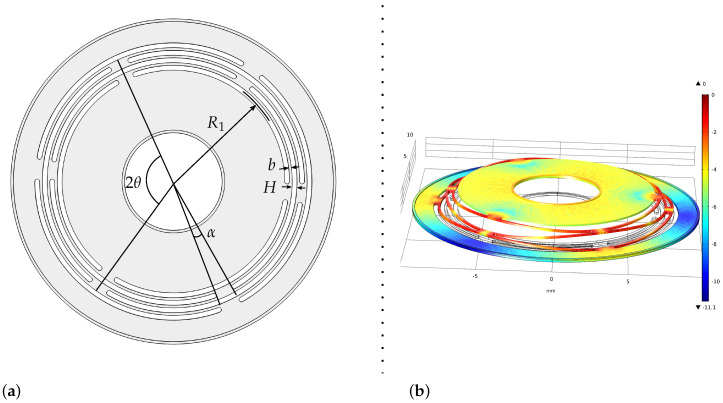
(**a**) Top view of one ortho-planar spring and parametrization. *H* is the spacing between the beams, *b* is the width of the beams in the radial direction. α is the aperture angle of the material remaining between two cuts and θ is half of the aperture angle of the cuts; (**b**) Normalized stress log(σ/max(σ)). A total force of 1 N was applied to the interior border. The maximal stress is located at the ends of the curved beams.

**Figure A2:**
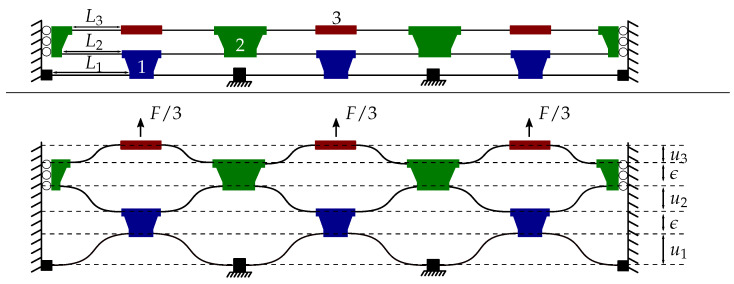
Sketch representing the unfolded ortho-planar springs. This 2D hyper-guided structure is assumed to be equivalent to one 3D ortho-planar spring under the assumption of a purely vertical motion (i.e., no radial displacement within the 3D structure). Tables 1, 2, 3 are the junctions between the beams. The hyper-guided system is presented at rest (**top**), and under a vertical force *F* (**bottom**).

**Figure A3:**
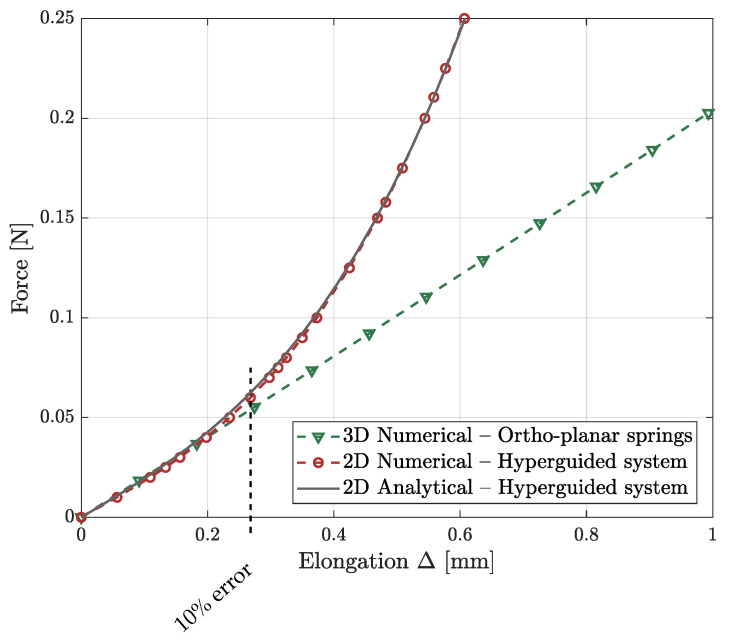
Comparison between the 3D finite element model of one ortho-planar spring and the 2D simplification based on the hyper-guided structure. The 2D simplification was solved numerically in COMSOL Multiphysics and with the analytical solution.

**Figure A4:**
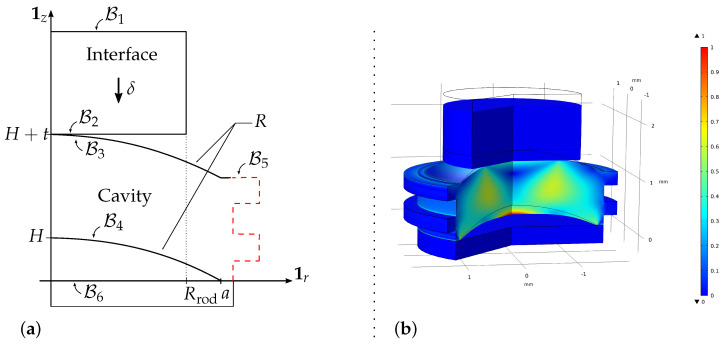
(**a**) Sketch of the domains and boundaries for the numerical model solving the compression of the cavity by the interface. The zero displacement on boundary $B_{5}$ is indicated by the dashed line. (**b**) Example of the axisymmetric model solved for δ=0.2 mm: normalized stress σ/max(σ).

**Figure A5:**
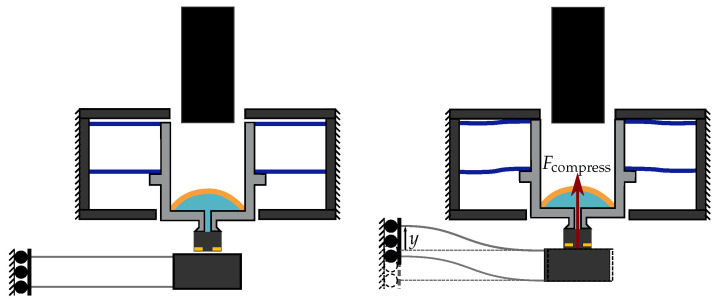
The module for the compression test. A table guided by two parallel beams compresses the component; therefore, applying a known pressure on its surface. This module mimics the industrial electrical testers. In contrast to simple passive capillary grippers, here, the meniscus was sucked into the cavity when the gripper moved up. Performing the compression test in this configuration allowed testing the component without squishing the liquid meniscus, which would result in the total loss of the self-alignment capability. In the case of our gripper, the liquid meniscus could be generated again in the next steps of the process, even though the component has been compressed.

## Data Availability

The data used to support the findings of this study are available from the corresponding author upon request.

## Supplementary Materials

The following supporting information can be downloaded at: https://www.mdpi.com/article/10.3390/mi13081323/s1. Video S1: Elongation of a glass specimen. Video S2: Proof of Concept. File S1: SupplMaterial Scaling system.

## Appendix A. Modeling of the Subsystems

This appendix presents the modeling of the subsystems composing the capillary gripper, namely the ortho-planar springs and the silicone cavity. Both subsystems were modeled using a finite element method that includes geometrical non-linearities. Additionally, we suggest here a 2D simplification of the ortho-planar springs. An analytical solution is presented, and its validity range is discussed.

### Appendix A.1. Ortho-Planar Springs

#### Appendix A.1.1. Presentation

The ortho-planar springs are mounted in parallel on the shell. At their center, the gripper’s core is pushed by the vertical load exerted by the actuator’s interface. Given the purely vertical solicitation of the actuator’s interface coupled to the high radial stiffness of the ortho-planar springs, the gripper’s core is guided in a vertical motion. In this configuration, one spring can be isolated and studied. Figure 2b shows the configuration of the two ortho-planar springs in parallel, for illustration purposes. There, the gripper’s core was replaced by a cylinder, on top of which the load was applied, while the outer boundaries of the springs were clamped on the shell (not represented).

#### Appendix A.1.2. Numerical Model

The stiffness of ortho-planar springs was obtained by a 3D finite element analysis developed with COMSOL Multiphysics and the Structural Mechanics Module, allowing geometrical non-linearities.

In this model, the exterior boundary is fixed, while an axial force *F* is applied to the inner boundary. The structure is modeled considering a linear elastic material description. It is left free to deform, except for the boundaries mentioned before.

The model was solved in the configuration mentioned here but also with a radial load, to compute the radial rigidity. Figure A1b shows a result for the model solved with a vertical force of 1 N applies on one ortho-planar spring. The numerical model of the spring was used as a basis for a discussion on the downscaling of the system, which can be found in the supplementary materials.

#### Appendix A.1.3. Analytic Simplification—Hyper-Guided System

With two ortho-planar springs in parallel, a pure translational motion is ensured. One can assume that the ortho-planar springs undergo a vertical motion only. Based on this hypothesis, an analytical simplification for the ortho-planar springs holding an axial load is proposed. The range of validity for this hypothesis will be discussed later.

Note that although the two ortho-planar springs in parallel are required for the purely vertical motion assumption, only one spring can be modeled to study the whole subsystem.

In this model, the circular ortho-planar spring structure is unfolded (and spread in the vertical direction by a length 2ϵ for visualization purposes only). ϵ is zero in practice. The system is presented in Figure A1.

Unfolding the structure yields three rows of straight beams (see Figure A2). Their lengths $L_{1}>L_{2}>L_{3}$, which are represented in Figure A2, are:

(A1) $$L_{i}=\left(R_{1}+\frac{i+1}{2}\left(b+H\right)\right)\left(\theta -\alpha \right),\;i\in \left\{1,2,3\right\}.$$

The geometrical definition of $R_{1}$, *b*, *H*, θ, and α can be found in Figure A1a: *b* is the width of the beams, *H* is the radial gap between the beams, and $R_{1}$ is the radius of the first cut. α represents the aperture angle of the material remaining between two cuts and θ is half of the aperture angle of the cuts.

Each table i∈{1,2,3} holds a total load F/3 under which its two beams of length $L_{i}$ deflect, giving a total deflection Δ. It writes [21]:

(A2) $$\begin{array}{cc} \frac{F}{3} & =\frac{3btEu_{i}^{4}}{3u_{i}L_{i}^{3}-\sqrt{6}tL_{i}^{3}\tanh\left(\sqrt{\frac{3}{2}}\frac{u_{i}}{t}\right)},\;i\in \left\{1,2,3\right\}, \end{array}$$

(A3) $$\begin{array}{cc} \Delta & =\sum_{i=1}^{3}u_{i}, \end{array}$$

where *t* is the thickness of the beams, *E* is the Young’s Modulus and $u_{i}$ is the displacement of row *i*.

Δ being known, solving the system of four Equations (A2) and (A3) yields *F*, along with the other variables $u_{1}$, $u_{2}$, $u_{3}$.

#### Appendix A.1.4. Discussions

Figure A3 compares the results of the 3D modeling of the ortho-planar springs and the 2D hyper-guided system. The finite element representation of the 2D model presented in Figure A2 was also modeled in COMSOL. Its solution is plotted together with the analytical solution: both computations yielded very similar results (see Figure A3). Indeed, the order of magnitude for the strain was $10^{-3}$, which remained compatible with the linearity assumption made to obtain in Equation (A2).

The 3D numerical model and its 2D simplification show good agreement for small deformations. For the dimensions chosen for designing the gripper, relative errors remain below 10% until a total elongation Δ=0.268 mm. The 2D simplification can be used for a pre-dimensioning step if the elongation remains small. However, due to the large elongations we can produce with our glass micro-system, the use of the 3D finite element simulation is required to catch the full behavior of the springs.

For large elongations, we explain the discrepancy between the 3D model’s solution and its 2D simplification as follows. As the displacement Δ increases, the traction in the beams increases. For the 2D hyper-guided system, this results in a strong increase in the stiffness, while for the curved beams of the actual 3D ortho-planar system, this extra load results in a radial motion directed towards the center. This relieves the stress in the beams, allowing a larger vertical elongation. Therefore, dimension *b* should be chosen carefully to ease this radial displacement. However, a too-small width *b* would result in a reduction of the radial stiffness, which is undesirable as it ensures the proper guidance of the gripper’s core.

### Appendix A.2. Numerical Modeling of the Silicone Cavity

To provide precise dimensioning and a deeper understanding of the system, the cavity was numerically modeled in COMSOL Multiphysics (Structural Mechanics module) [22].

In this model, the solid elastic domain of the cavity is an axisymmetric structure. The rigid domain of the vertical actuator’s interface applies a force to the cavity. The contact area between the parts changes and grows as the height of compression δ increases.

The domains are assumed to be made of linear elastic material. The cavity is made of a tin cured silicone rubber. Its hardness is 40 on the Shore A hardness scale. As a first guess for the Young’s modulus, we used the empirical model from the study by Qi et al. [23]. The relationship between the durometer hardness of elastomeric materials on the Shore A scale and the Young’s modulus of the material can therefore be approximated by $E=10^{0.0235S-0.6403}$ MPa, where E is the Young’s modulus of the elastomeric material, and S=40 is the Shore A hardness. Using this formula, the Young’s modulus of our silicone rubber can be approximated by E = 1.99 MPa. Its Poisson coefficient is 0.49.

Figure A4a presents the modeled geometry. The displacement δ of the vertical actuator is transmitted to the modeled system through boundary $B_{1}$. It results in its contact with the cavity, i.e., between boundaries $B_{2}$ and $B_{3}$.

$B_{4}$ is left free to deform until it contacts the boundary $B_{6}$. The clamping condition on $B_{6}$ and $B_{5}$ (the shape of which came from the molding of the membrane inside the gripper’s core), reads ${\left.u\right|}_{B_{5}}={\left.u\right|}_{B_{6}}=0$, where $u=u_{r}1_{r}+u_{z}1_{z}$ is the displacement field in the domains.

This static model is solved using a continuation method on the compression parameter δ, such that, at initialization, the displacement field in the domains at the (k + 1)th value of δ is [22]:

(A4) $$u_{\mathrm{init}}^{k+1}=u_{\mathrm{sol}}^{k}+\frac{\partial u_{\mathrm{sol}}^{k}}{\partial \delta }\left(\delta \left(k+1\right)-\delta \left(k\right)\right),$$

where $u_{\mathrm{sol}}^{k}$ is the displacement field resulting from the model solved for the kth value of δ. The result of the solving for δ=0.2 mm can be seen in Figure A4b.

## Appendix B. Compression of Components

This appendix presents the working principle of the compression module, displayed in Figure A5.

Once the compression module is in contact with the handled component, a pressure $P_{\mathrm{compress}}$ is applied by moving up the left ends of the beams. The displacement *y* required to apply the pressure is [21]:

(A5) $$y=\frac{SP_{\mathrm{compress}}}{k_{\mathrm{module}}}=\frac{F_{\mathrm{compress}}}{k_{\mathrm{module}}},\;\mathrm{where}\;k_{\mathrm{module}}=\frac{24EI}{L^{3}},$$

where *S* [m${}^{2}$] is the surface of the component and $k_{\mathrm{system}}$ [N·m${}^{-1}$] is the stiffness of the guided compression system. E [Pa] and I [m${}^{4}$] are, respectively, the Young’s modulus of the beams and the quadratic moment of area. *y* must be much lesser than *L*.
