# Supplementary material for: A Volume-Tuning Capillary Gripper That Enhances Handling Capabilities and Enables Testing of Micro-Components
Source: Micromachines (Basel). 2022 Aug 16;13(8):1323. doi: 10.3390/mi13081323 (PMC9412535; doi:10.3390/mi13081323)
Supplement: Supplementary file 1 [file micromachines-13-01323-s001.zip › File S1. SupplMaterial_Scaling_system.pdf]

A Volume-Tuning Capillary Gripper that Enhances Handling Capabilities and Enables Testing of Micro-Components

Supplementary materials

Adam Chafaï[1], Amin Ibrahimi[2], and Pierre Lambert[1]

[1] Transfers, Interfaces and Processes (TIPs) department, CP 165/67, 50 Av. F.D. Roosevelt,
Université Libre de Bruxelles (ULB), 1050 Brussels, Belgium;
[2] Vrije Universiteit Brussel (VUB), Bd. de la Paine 2, 1050 Brussels, Belgium;

Scaling the system down

This section will shortly discuss the scaling of the ortho-planar springs . If considering components of different sizes, the scaling of the gripper can be achieved by modulating the surface of its picking head in a wide range of sizes and shapes, to better suit the component to handle.

However, here, we will focus more strongly on the downscaling of the system's footprint. The ortho-planar springs being the limiting parts, as their maximal elongation and rigidity come from their large radius and small thickness, one should seek the configuration where the radius could be minimized, and the mechanical properties could be preserved.

A numerical study was performed to compare the different configurations of radial and axial downscaling in terms of maximal stress, elongation at rupture, and axial stiffness. The radial dimension was changed to 75%, 50%, and 25% of the original size (outer radius 10 mm). The thickness (axial scaling) was varied between 10% and 100% of the original value used for the proof of concept (thickness 0.144 mm).

Figure 1a presents the overall result of the study but is mainly meant to visualize the evolution of the stress with the axial and radial downscaling. There, the four surfaces show the radial scaling variations. The vertical axis represents the ratio between the stress (Tresca criterion) and the ultimate stress $\sigma_{\max}$, to which a safety coefficient $c_s = 3$ is applied. The stress considered for the rupture is $\sigma_{\max} = 2.1$ GPa. This value has been obtained from destructive tests of glass flexure hinges and by numerically computing the stress for the configuration where the rupture occurs. The use of flexure hinges for the rupture tests allows a better assessment of the ultimate stress, as these structures are much simpler than the ortho-planar springs in terms of modeling, manufacturing, and experimental elongation tests, which limits the number of sources of errors.

In Figure 1a, one outer diameter is given by one surface, and within one surface, the thickness of the ortho-planar spring is given by the axial downscaling parameter (the reference 1:1 has a thickness of 0.144 mm).The elongation can be obtained on the third axis. As expected, the stress increases as the axial elongation Δ and the thickness increase. The radial scaling also plays a strong role here. The cut off of the vertical axis is at $\sigma/(\sigma_{\max} c_s) = 1$, which represents the rupture.

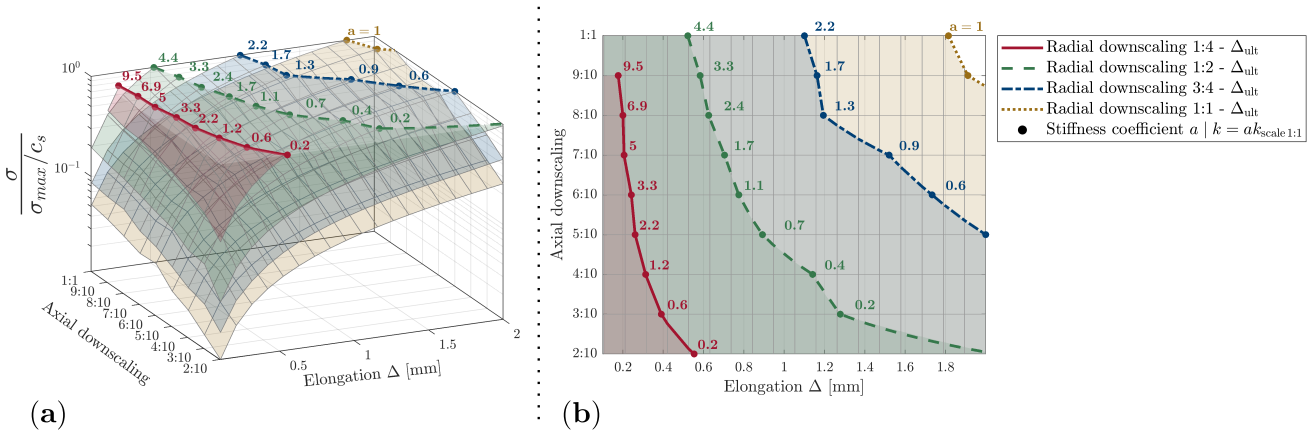

Figure 1: **a** Results of the downscaling study for the ortho-planar springs. The stress $\sigma/(\sigma_{\text{max}}c_s)$ plotted against three parameters: the axial elongation, the axial and the radial downscaling factors. c_s is the safety coefficient. The graph presents a fourth dimension through the coefficient a. a is the number of ortho-planar springs to stack in order to reach the stiffness of one original ortho-planar spring (scaling 1:1). The fifth dimension is represented by the plain and dashed lines, which show the ultimate elongation Δ_{ult} for the considered configurations; **b** Projection in the plane $\sigma/(\sigma_{\text{max}}c_s) = 1$. The reference configuration of radial and axial downscaling 1:1, for which a=1 has been manufactured and used for the proof of concept.

The intersection of these surfaces with the horizontal plane $\sigma/(\sigma_{\text{max}}c_s) = 1$ yields curves representing the axial elongations at rupture. In Figure 1b (the projection of Figure 1a in the horizontal axes), this elongation is given by the plain and dashed lines. One can see that only a few configurations allow an elongation as large as our original proof of concept does (radial and axial downscaling 1:1). However, as the vertical motion is expected to be below 1 mm, any configuration with an ultimate elongation above 1 mm could be considered. The 25% downscaling configurations seem not to be to considered here, at least with such a large safety coefficient. More generally, equivalent configurations in terms of ultimate elongations can be obtained from this graph.

In addition, the coefficients a reflect the evolution of the stiffness with the radial and axial downscaling. $1/a$ is the number of ortho-planar springs to stack in order to reach the stiffness of one original ortho-planar spring (scaling 1:1). $a < 1$, therefore, stands for lower stiffness configurations, which, at constant radial scaling, occur for a decreasing thickness. At a constant thickness, a increases as the springs are radially scaled down and would exceed 1 once the spring becomes stiffer than the original configuration. In a practical situation, $a \leqslant 1$ seems beneficial to avoid too large parasitic droplets. However, although the dynamical behavior of the system has not been studied here, a stiffer system would be beneficial in terms of dynamical response, as it would result in a gripper's core moving up faster, although the extra stiffness should be compensated by an even stiffer cavity.
